# LIKELIHOOD OF ADOPTION OF CAREMOBI: ADDRESSING COMMUNICATION GAPS BETWEEN ADULT DAY HEALTH CENTERS AND PRIMARY CARE

**DOI:** 10.1093/geroni/igac059.3015

**Published:** 2022-12-20

**Authors:** Zuha Ali, Tina Sadarangani

**Affiliations:** Indiana University School of Medicine, Indianapolis, Indiana, United States; New York University, New York, New York, United States

## Abstract

Adult Day Health Centers (ADHC) are non-residential congregate facilities that serve 250,000 cognitively and functionally impaired adults daily. These centers are effective platforms for chronic disease management and interdisciplinary care coordination, with serial observations on participants over multiple hours and days per week. However, previous research indicates that ADHCs experience challenges in communication with primary care providers (PCPs). This is because 92% of ADHCs lack interoperable EHRs (electronic health record systems) and do not have the financial and technical infrastructure to implement them. CareMOBI (Mhealth for Organizations to Bolster Interconnectedness) is a mobile application in development meant to streamline the information communication between ADHC, PCP, and family caregiver populations. The purpose of this study was to understand the likelihood of adoption of CareMOBI by primary care providers. Demographics of the study included 87.50% female and 12.5% male primary care providers with a median age of 38 years. Participants were recruited through parallel convergent design, and data was merged into a matrix defined by 4 major themes: perceived value in geriatric care, ease of use, fit within workflow, and likelihood of adoption. The results revealed that a majority percentage of providers were likely to adopt the app, saw the app as valuable, and easy to use. In response to fit within a provider’s workflow, apprehensions surrounding interoperability arose. These apprehensions regarding interoperability are the most important to address through a user-centered approach and as the app is pilot tested into adult day health centers.

